# Supplementary material for: Ubiquitin-specific protease 21 stabilizes BRCA2 to control DNA repair and tumor growth
Source: Nat Commun. 2017 Jul 26;8:137. doi: 10.1038/s41467-017-00206-2 (PMC5526993; doi:10.1038/s41467-017-00206-2)

File Name: Supplementary Information
Description: Supplementary Figures and Supplementary Tables

File Name: Peer Review File
Description:

Primer Name	Sequence (5'-3')	AT (°C)
**RT-PCR**		
USP21 F	5'-TTCCCATGGCTCCTTCCACATGAT-3'	58
USP21 R	5'-GTTTCGAAGGCCAACATGACCAGA-3'	58
BRCA2 F	5’-CAGTGGTATGTGGGAGTTTGT-3’	58
BRCA2 R	5’-ACCTCAGCTCCTAGACTTTCA-3’	58
Rad51 F	5’-TGGCCCACAACCCATTT-3’	58
Rad51 R	5’-GCAACAGCCTCCACAGTAT-3’	58
PALB2 F	5’-GGAAGAAGTCACCTCACACAA-3’	58
PALB2 R	5’-GCACTATTACTCCAAGAAAGGGA-3’	58
GAPDH F	5’-GGAGTCAACGGATTTGGTCG-3’	58
GAPDH R	5’-GAGGCATTGCTGATGATCTTG-3’	58
RPS16 F	5’-AAACGCGGCAATGCTCTCATCAAG-3’	58
RPS16 R	5’-TGGAGATGGACTGACGGATAGCAT-3’	58
**CRISPR screening**		
Primer A F	5’-ACTGAGCCAACCACCTAATG-3’	61*
Primer B R	5’-GAGAAGTGCTACGTCTCAAAGT-3’	61*
Primer C R	5’-GGACCGAAATGGCACACCATG-3’	61*

Supplementary Table 1. Primer sequences
AT: annealing temperatures; * touchdown from 71 °C.

shRNAs	Sequence (5'-3')
sh-U21-1	5'-CCACTTTGAGACGTAGCACTT-3'
sh-U21-2	5'-GCCTTTCTACTCTGATGACAA-3'
sh-Luc	5’-ATGTTTACTACACTCGGATAT-3’
**siRNAs**	**Sequence (5'-3')**
si-USP21-1	5’-CCACCCACTTTGAGACGTA-3’
si-USP21-2	5’-GCCTTTCTACTCTGATGACAA-3’
si-53BP1	5’-GCTATATCCTTGAAGATTT-3’
si-BRCA2	ON-TARGET smart-pool (Dharmacon)
si-control	ON-TARGET smart-pool (Dharmacon)
**guideRNAs**	**Sequence (5'-3')**
U21-1	5’-GCCTGTGGTGTCCACAATGCCCC-3’
U21-2	5’-AGGACCGGGCGGATATCATG-3’
U21-3	5’-AAGACAGCTCGGAATCGAGT-3’

Supplementary Table 2. siRNA/shRNA and guideRNA sequences

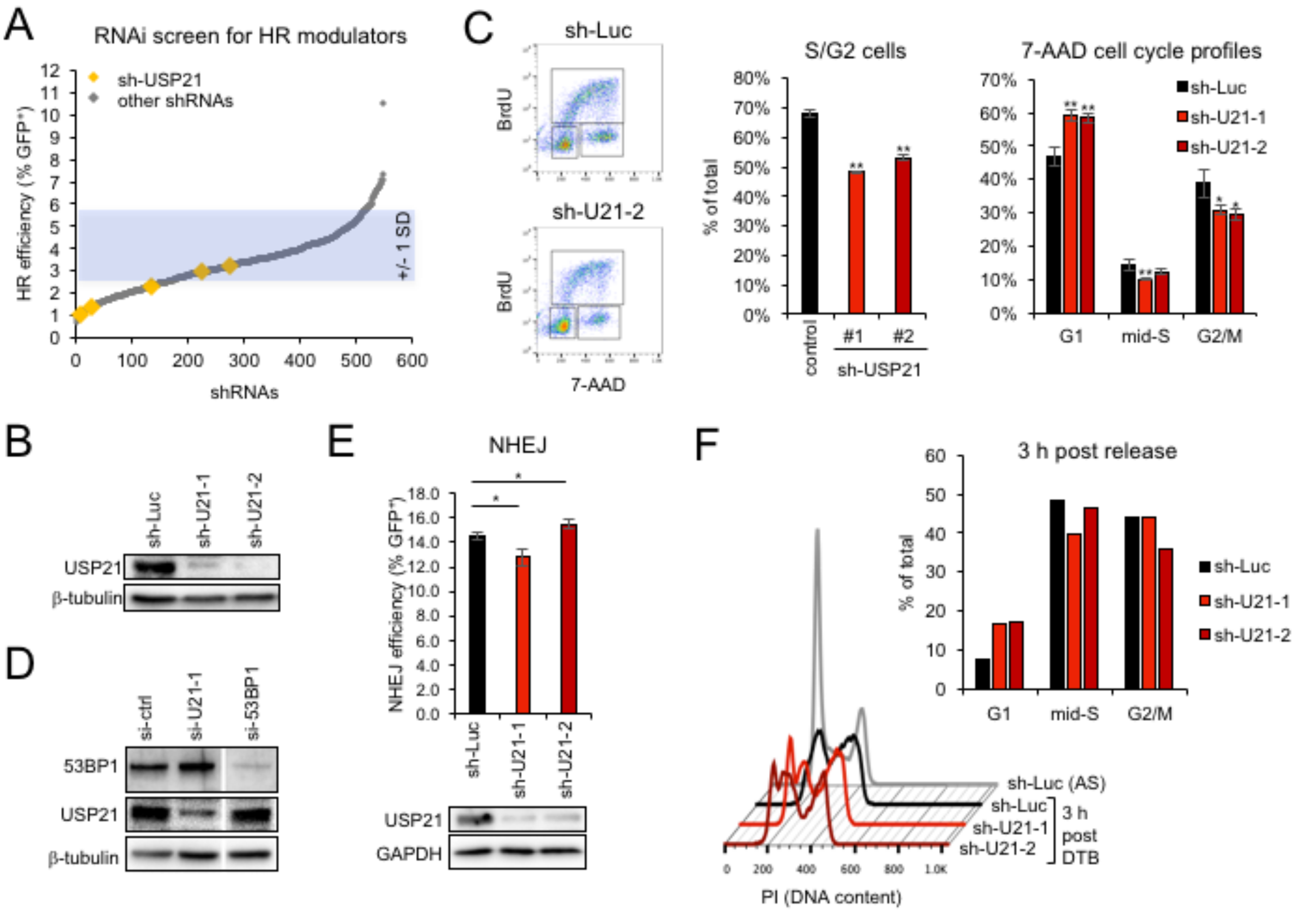

Supplementary Figure 1. HR screen identifying USP21, USP21 knockdown efficiency, and cell cycle profile upon USP21 depletion. (A) RNAi screen to measure HR efficiency in TRI-DRGFP U2OS cells stably transduced with shRNAs from a chromatin-focused RNAi library. HR was measured as % GFP$^+$ cells; yellow diamonds represent USP21-specific shRNAs, grey diamonds represent other shRNAs included in this subset of the screen (see Khurana et al., Cell Reports 2014 for details). (B) Western blot analysis for USP21 and ß-tubulin in U2OS-HR cells expressing the indicated shRNAs. (C) Cell cycle distribution in the presence or absence of USP21 knockdown in TRI-DRGFP U2OS cells. Representative FACS analyses of BrdU incorporation are shown. DNA content was measured using 7-AAD. S/G2 cells comprise BrdU$^+$ and 7-AAD high (2 n) cells. Cell cycle subsets were further defined based on DNA content as G1 (1 n), mid-S and G2/M (2 n). Samples were analyzed in triplicate. Values are expressed as mean and s.d. (D) Western blot analysis for USP21, 53BP1 and ß-tubulin in U2OS-NHEJ cells expressing the indicated siRNAs. (E) NHEJ efficiency in U2OS-NHEJ cells expressing the indicated shRNAs. Samples were analyzed in triplicate. Values are expressed as mean and s.d. A Western blot for USP21 knockdown efficiency is shown. (F) Cell cycle profiles in cells expressing the indicated shRNAs in the presence or absence of double-thymidine block (DTB). Propidium Iodide (PI) staining was performed in asynchronous (AS) cells or 3 h post DTB, DNA content was analyzed as in (D). Similar results were obtained 1 h post DTB (not shown).

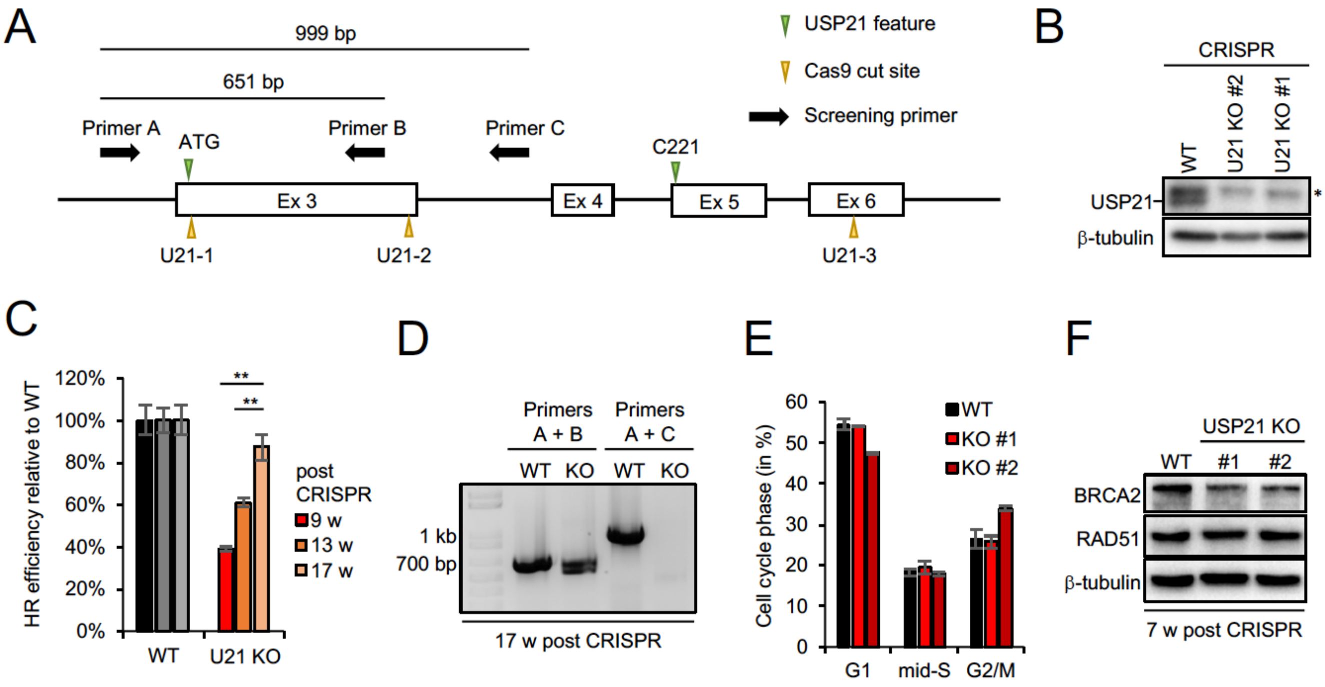

Supplementary Figure 2. Generation and analysis of USP21 KO cells using CRISPR/Cas9. (A) Schematic of CRISPR/Cas9 strategy for USP21 gene inactivation. Cells were transfected with a combination of the indicated guide-RNAs (gRNAs); (partial) deletion of intervening DNA was analyzed using indicated primer pairs (A+B or A+C), expected fragment sizes for the wildtype USP21 locus are shown. (B) Western Blot analysis of USP21 and ß-tubulin in parental DRGFP-U2OS control cells (WT) and two independent USP21 KO clones from Fig. 1B, asterisk indicates a non-specific band. (C) HR efficiency in a USP21 KO clone after the indicated times in culture. HR was measured as described in Fig. 1A. (D) Genomic PCR of WT cells and the USP21 KO clone from (C) after 17 weeks of culture. CRISPR/Cas9-mediated mutagenesis resulted in deletion of genomic DNA containing primer C on all alleles. Altered sizes of PCR fragments from primer pair A/B indicate additional genomic defects. (E) PI analysis of cell cycle subsets in WT and USP21 KO cells from Fig. 1B after 7 weeks in culture. (F) Western blot analysis of the indicated proteins in USP21 KO clones from Fig. 1B after 7 weeks in culture.

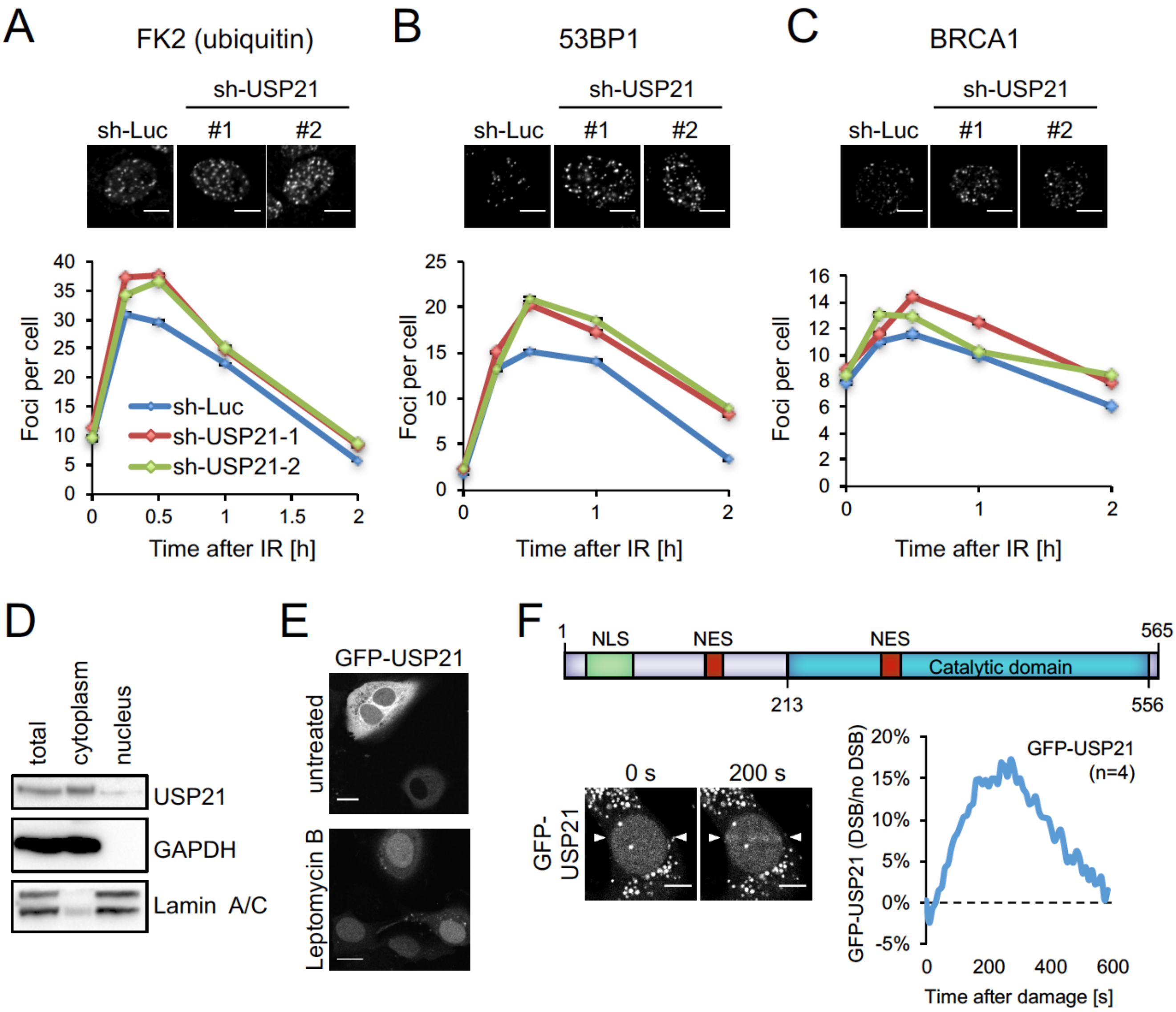

Supplementary Figure 3. USP21 loss promotes ubiquitination, 53BP1 and BRCA1 foci formation at DSBs. (A, B, C) Foci formation of poly-ubiquitin (FK2, A), 53BP1 (B) or BRCA1 (C) in DRGFP-U2OS cells in the presence or absence of USP21 knockdown. Cells were left untreated or irradiated with 5 Gray and incubated for indicated time frames. Images were analyzed using Opera high content imaging and Acapella 2.6 software. Scale bars, 10 μm. N > 300 cells per sample, representative images are shown. Values are expressed as mean and s.d. (D) USP21 western blot analysis of total, cytoplasmic and nuclear fraction from HEK293T cells. (E) Immunofluorescence analysis of U2OS cells transiently expressing GFP-USP21 in the presence or absence of leptomycin B (LMB) treatment. Scale bar: 10 μm. (F) Live cell imaging of U2OS cells transiently expressing GFP-USP21 following laser micro-irradiation in the presence of LMB pretreatment. Prior to DSB induction, cells were subjected to double-thymidine block, then released for 4 h. Representative images of 0 s and 200 s post DSB induction are shown, scale bars: 10 μm. A schematic of USP21 domains depicting nuclear localization (NLS) and nuclear export signals (NES) is shown.

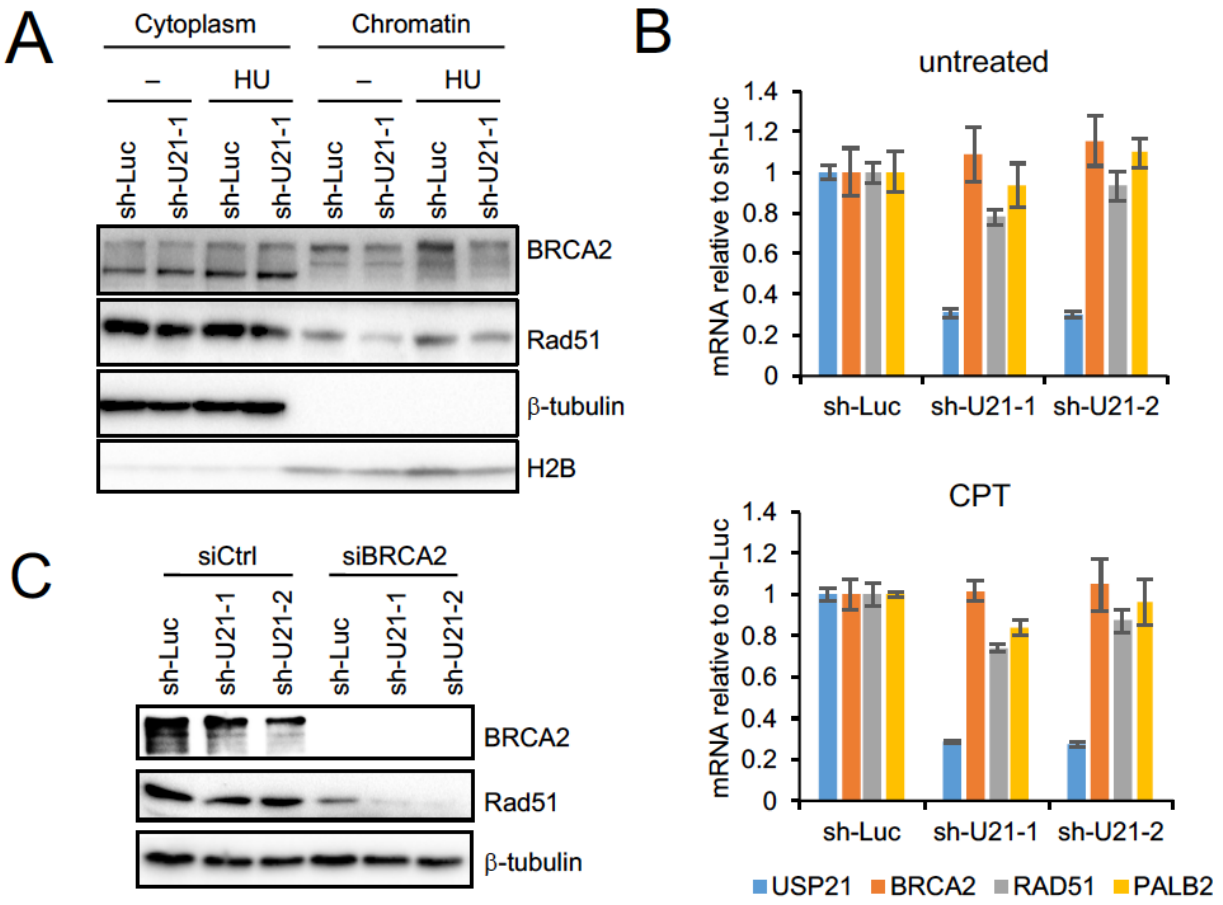

Supplementary Figure 4. Chromatin association and expression of Rad51 and BRCA2 upon USP21 loss. (A) Western blot analysis of the indicated proteins in cytoplasmic and chromatin-bound U2OS cell lysates. Cells were infected with the indicated shRNA and exposed to 2 mM HU for 24 h or left untreated. (B) Expression of Rad51, BRCA2 and PALB2 mRNAs normalized to GAPDH in U2OS cells in the presence or absence of USP21 knockdown. U2OS cells were either left untreated or treated with CPT for 1 h. Expression levels relative to sh-Luc are shown. Samples were analyzed in triplicate. Values are expressed as mean and s.d. (C) BRCA2 and RAD51 western blot analysis in U2OS cells expressing the indicated shRNAs together with control or BRCA2 siRNA.

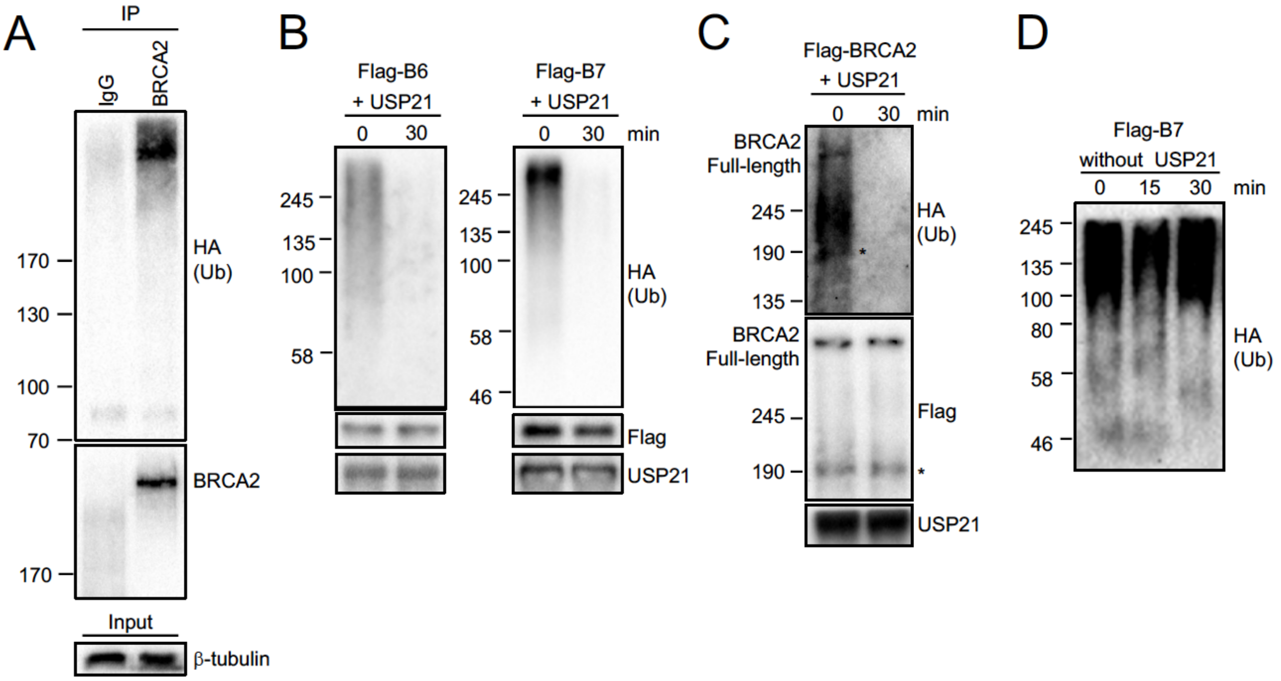

Supplementary Figure 5. USP21 deubiquitinates BRCA2 in vitro. (A) Denaturing IP of endogenous BRCA2 in HA-Ub-transfected HEK293T cells after 3 h of MG-132 treatment. Precipitates were probed with indicated antibodies, β-tubulin served as a control for input. Denaturing conditions efficiently disrupted endogenous BRCA2 complexes as no RAD51 protein was detected in the BRCA2 IP (not shown). (B) Western blot analysis of the indicated proteins after in vitro deubiquitination. Immuno-purified Flag-BRCA2-B6 and -B7 fragments were incubated with recombinant USP21 for 0 or 30 min. (C) In vitro deubiquitination analysis as in (B), using full-length Flag-BRCA2. Asterisk indicates BRCA2 degradation product. (D) In vitro deubiquitination of immuno-purified Flag-BRCA2-B7 as in (B), in the absence of recombinant USP21.

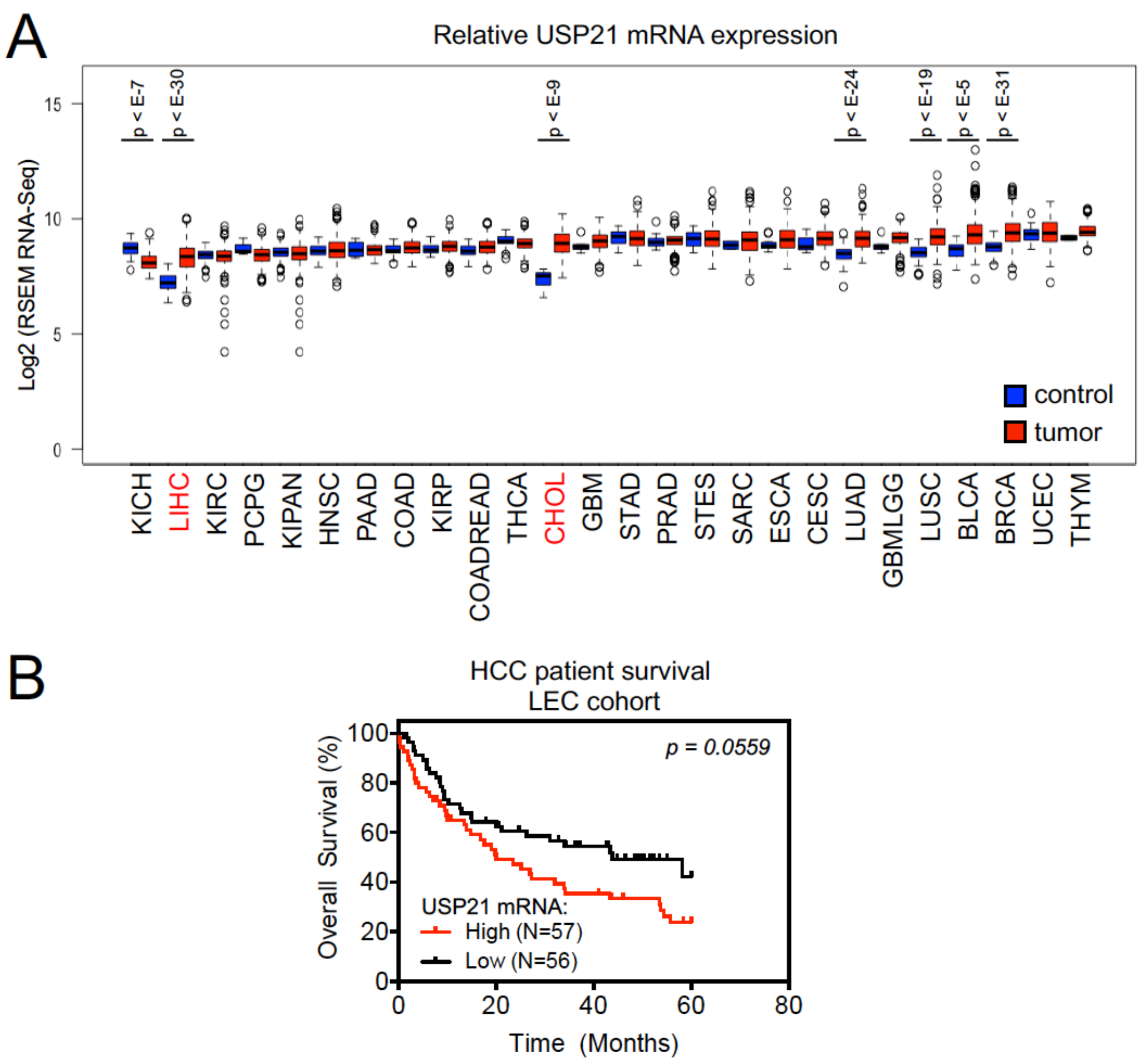

Supplementary Figure 6. USP21 expression across tumor types. (A) Relative expression across a cohort of 10,205 primary tumor samples from 26 different cancer types. Blue indicates normal tissue, red indicates primary tumor samples. Normalized USP21 gene expression log2 values across 26 cancer types are shown: glioblastoma multiforme (GBM), glioma (GBMLGG), head and neck squamous cell carcinoma (HNSC), kidney chromophobe (KICH), pan-kidney cohort (KICH+KIRC+KIRP) (KIPAN), kidney renal clear cell carcinoma (KIRC), kidney renal papillary cell carcinoma (KIRP), Liver hepatocellular carcinoma (LIHC), lung adenocarcinoma (LUAD), lung squamous cell carcinoma(LUSC), pancreatic adenocarcinoma (PAAD), pheochromocytoma and paraganglioma (PCPG), prostate adenocarcinoma (PRAD), sarcoma (SARC), stomach adenocarcinoma (STAD), stomach and esophageal carcinoma (STES), thyroid carcinoma (THCA), thymoma (THYM), uterine corpus endometrial carcinoma (UCEC). Increased USP21 expression was most pronounced in two liver tumors: LIHC and CHOL. The horizontal line in the boxplot depicts the median, bottom and top hinges of the box denote first and third quartile, respectively, whiskers represent +/- 1.5 IQR. (B) Kaplan-Meier survival analysis of 113 HCC cases from the LEC cohort , separated based on the level of USP21 expression in HCC samples as determined by microarray analysis (see Fig. 4D for details).

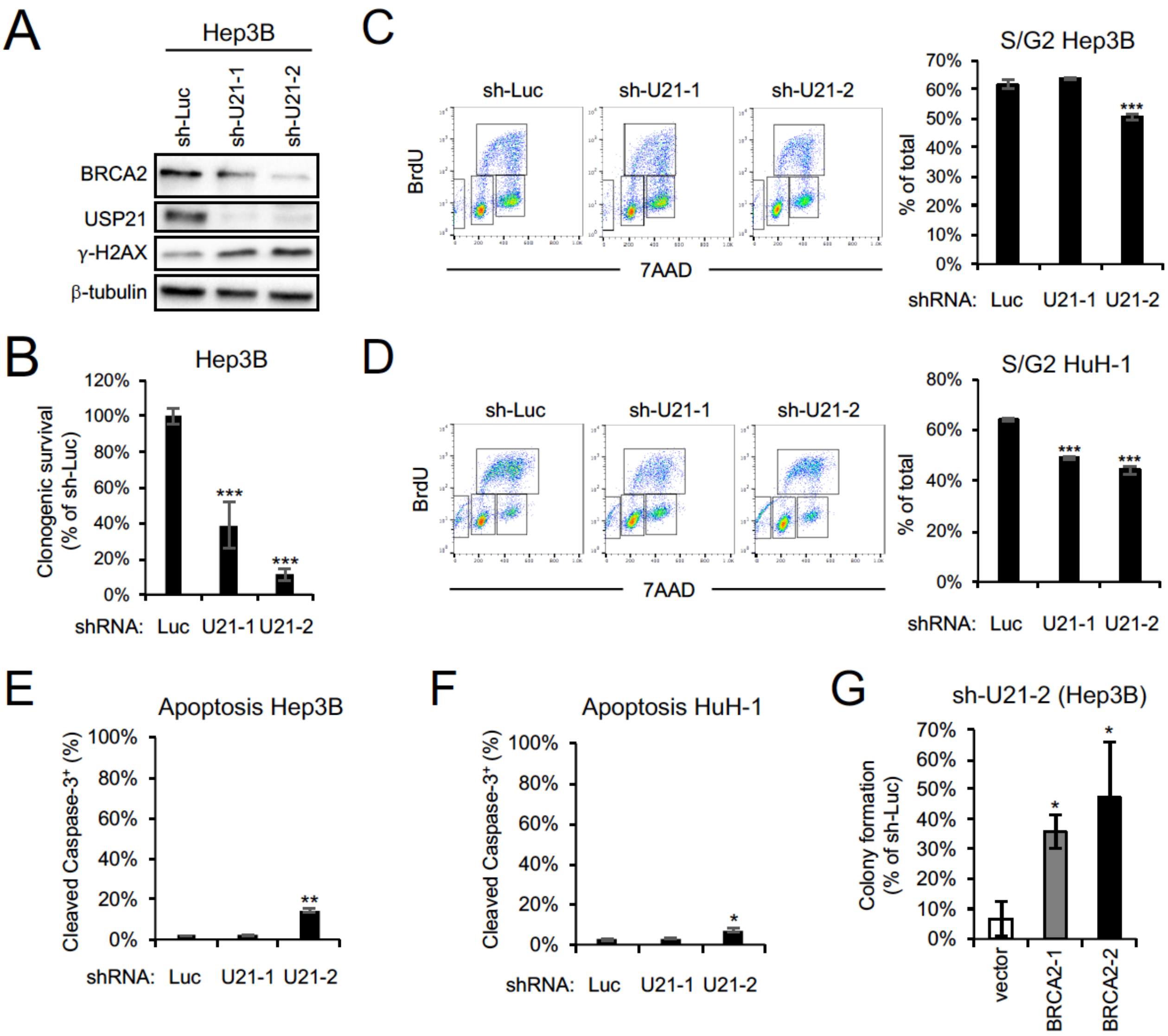

Supplementary Figure 7. Effects of USP21 loss in HCC cell lines. (A) Western blot analysis of the indicated proteins in the Hep3B hepatoma cell line in the presence or absence of USP21 knockdown. (B) Clonogenic survival of Hep3B cells in the presence or absence of USP21 knockdown. Samples were analyzed in triplicate and normalized to sh-Luc. Values are expressed as mean and s.e.m. from three independent experiments. (C, D) Cell cycle distribution in the presence or absence of USP21 knockdown in Hep3B (C) or HuH1 cells (D). Representative FACS analyses of BrdU incorporation are shown. DNA content was measured using 7-AAD. The fraction of $BrdU^{+}$ and/or 7-AAD high (2 n) cells is graphed as S/G2 cells. Samples were analyzed in triplicate. Values are expressed as mean and s.d. (E, F) Cleaved Caspase-3 assay in Hep3B (E) or HuH1 cells (F) in the presence or absence of USP21 knockdown. Samples were analyzed by FACS in triplicate. Values are expressed as mean and s.d. (G) Clonogenic survival after USP21 depletion in Hep3B cells stably transfected with either empty vector or Flag-BRCA2 (BRCA2-OE). The efficiency of colony formation in sh-USP21-2 cells was normalized to sh-Luc controls. Two independent BRCA2-OE clones are shown. Samples were analyzed in triplicate, values are expressed as mean and s.d.

Supplementary Figure 8. Uncropped Western blot images.

Figure 2A

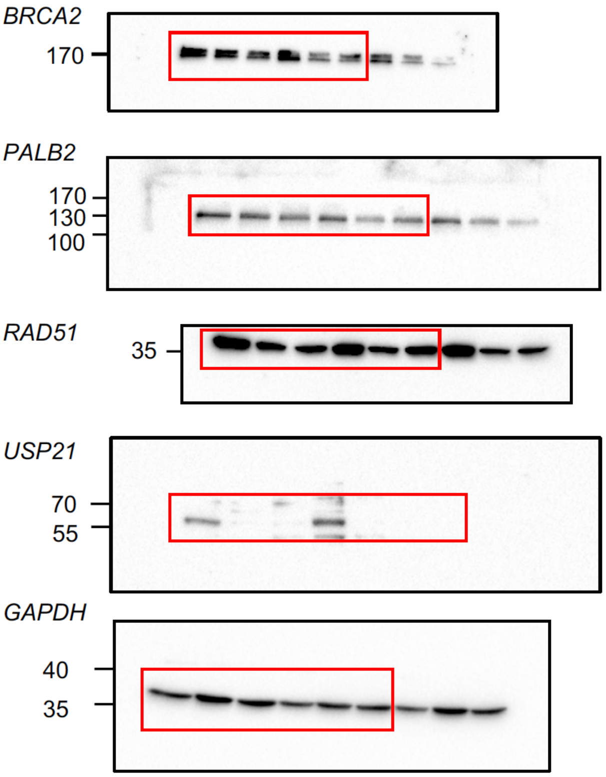

Figure 2B

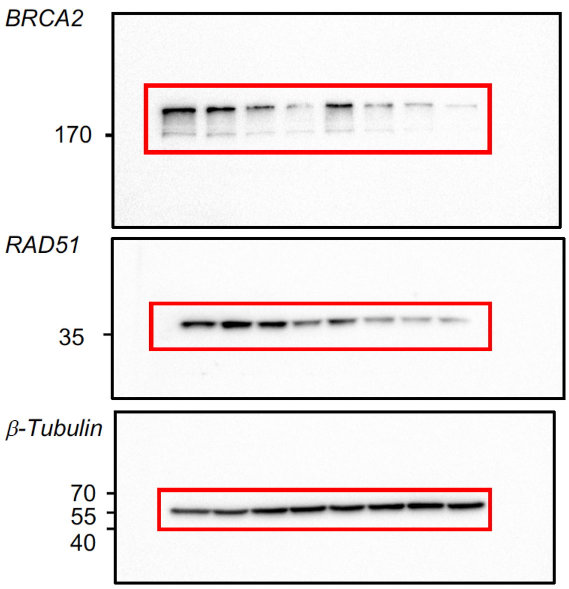

Figure 2C

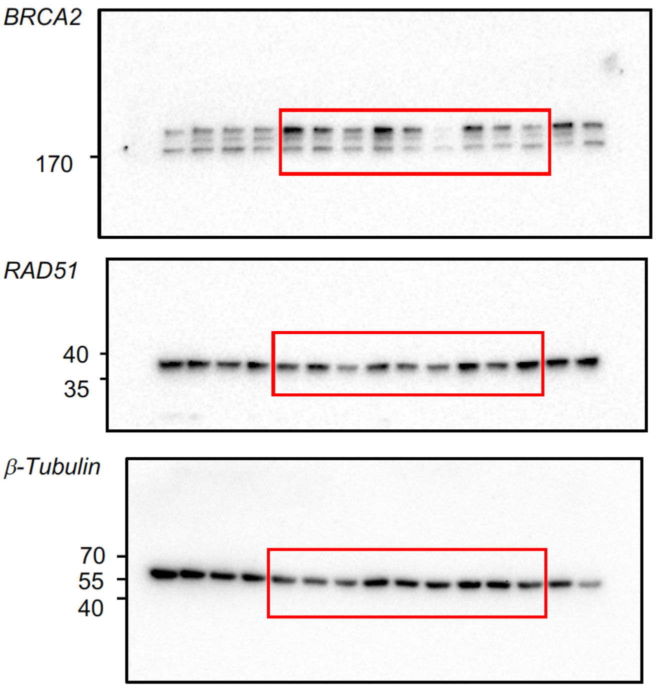

Figure 2D

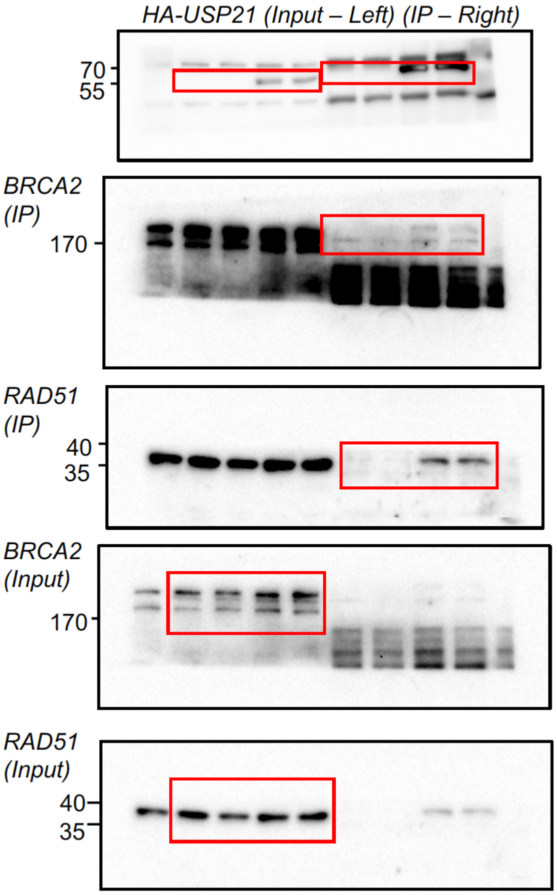

Figure 2E

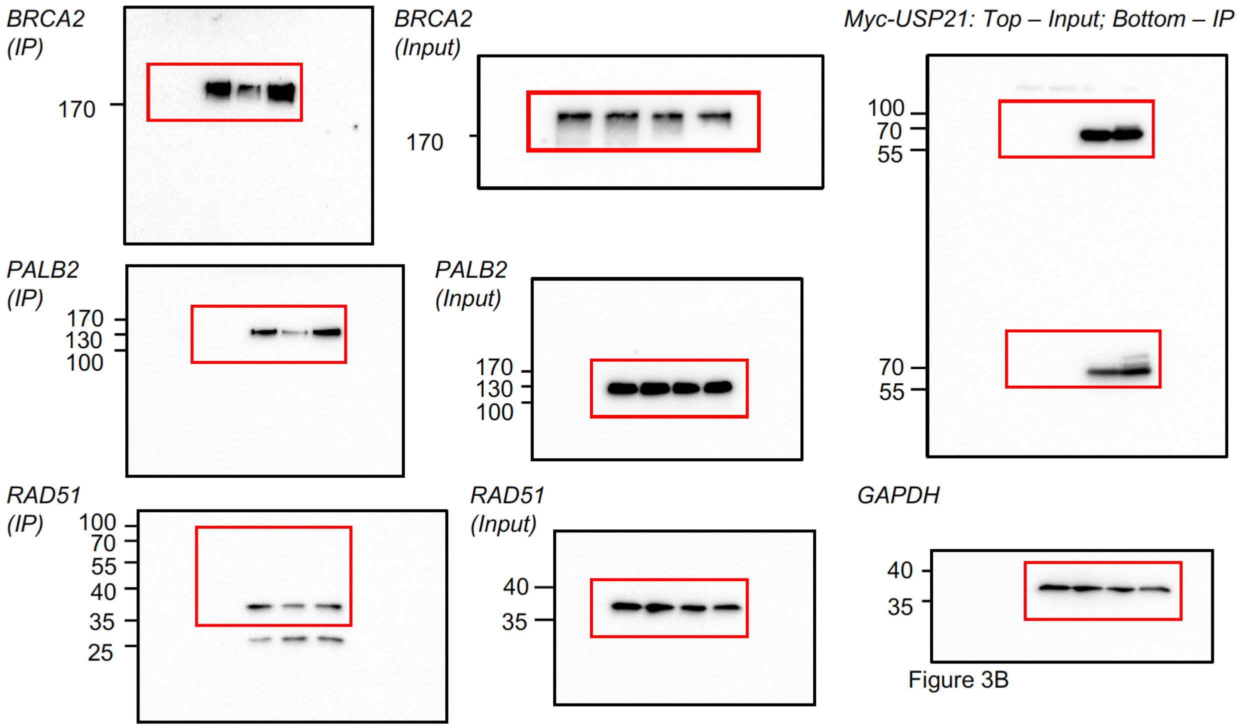

Figure 3A

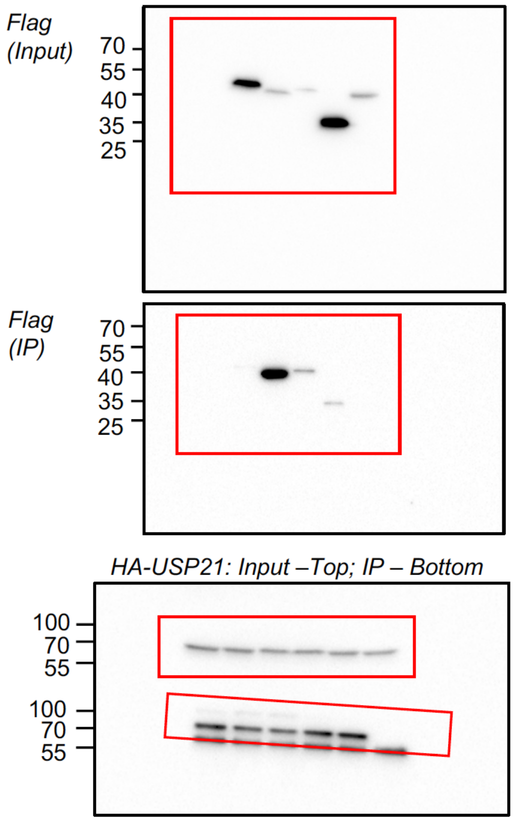

Figure 3B

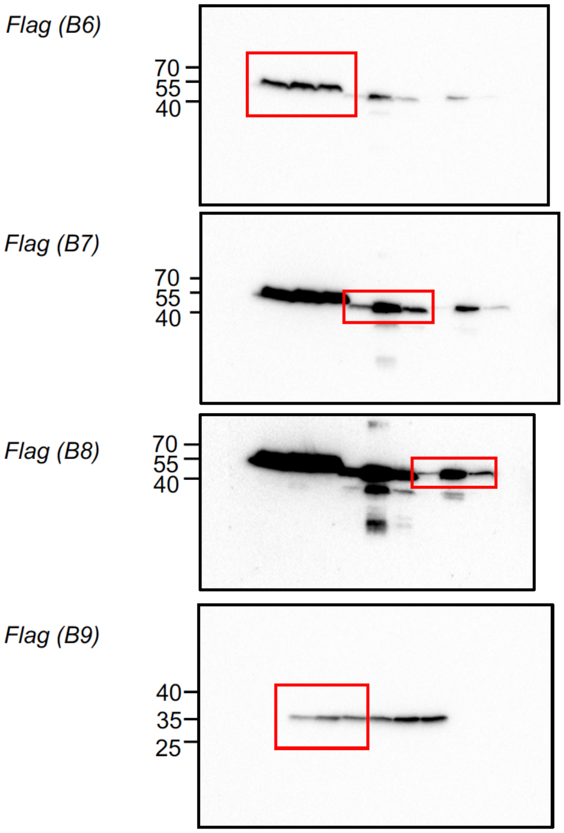

Figure 3B (continued)

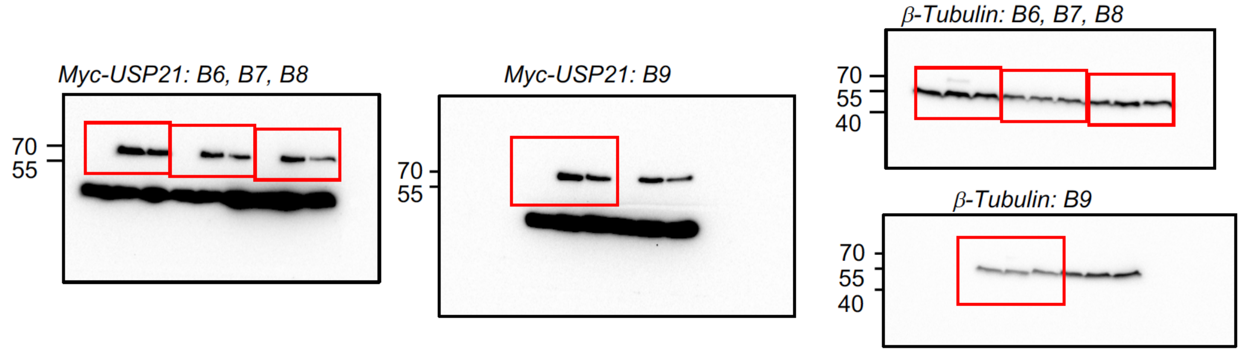

Figure 3C

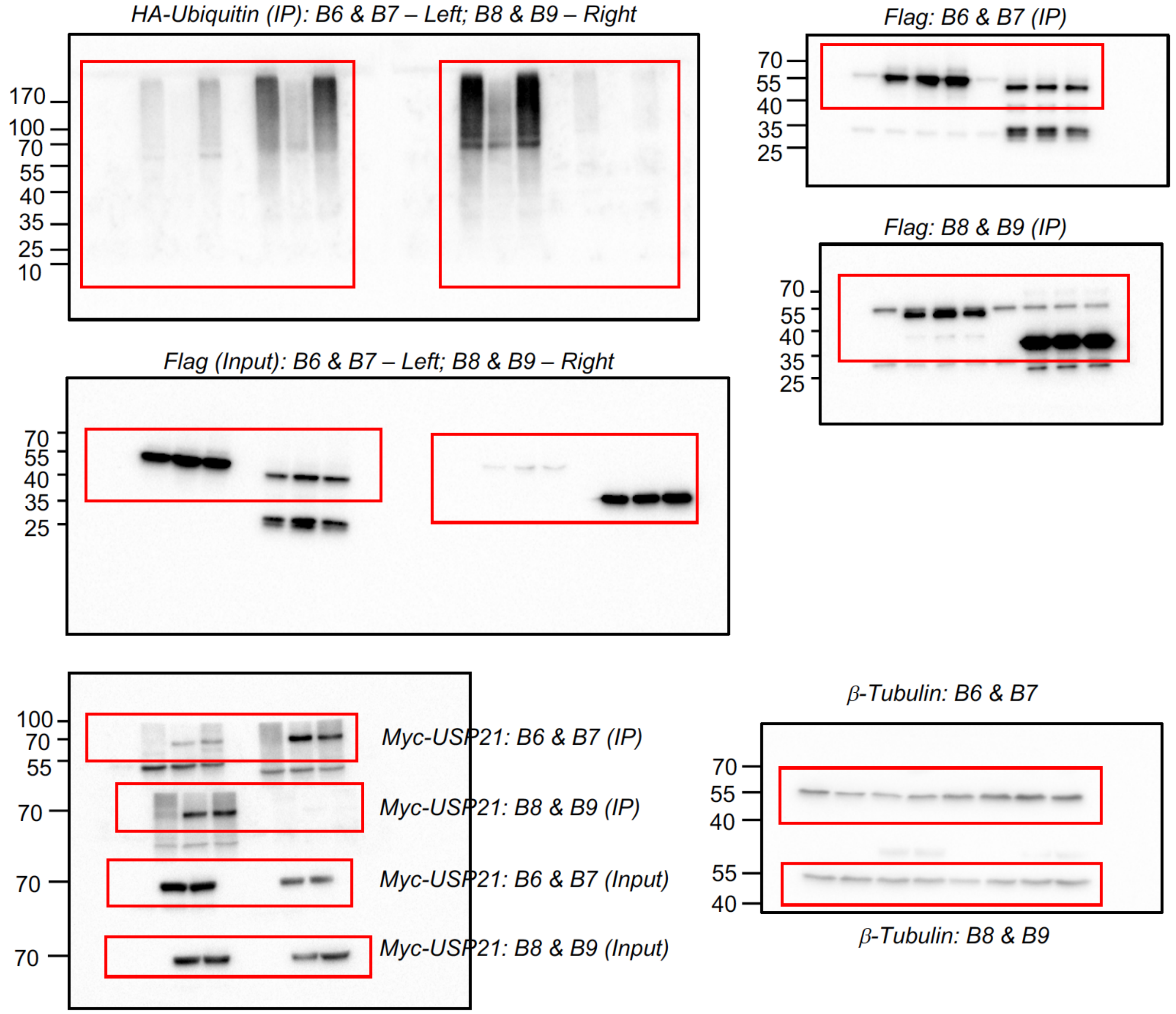

Figure 3D

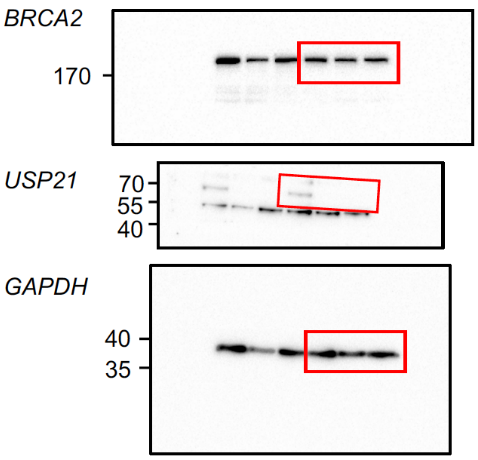

Figure 3E

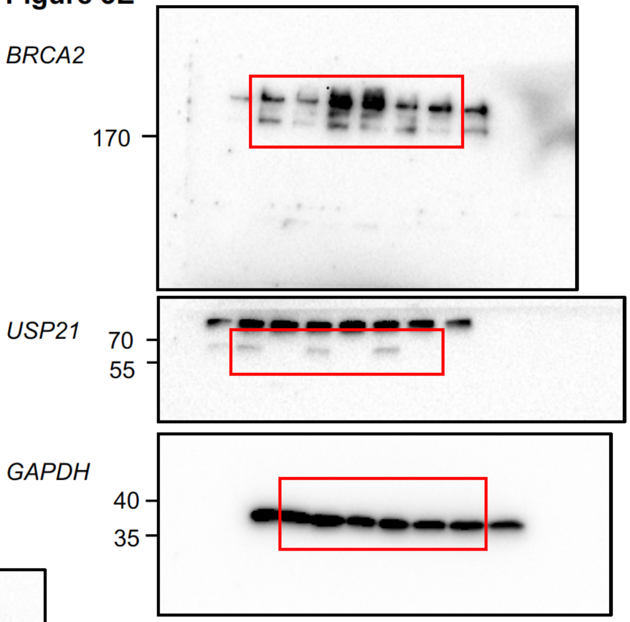

Figure 5A

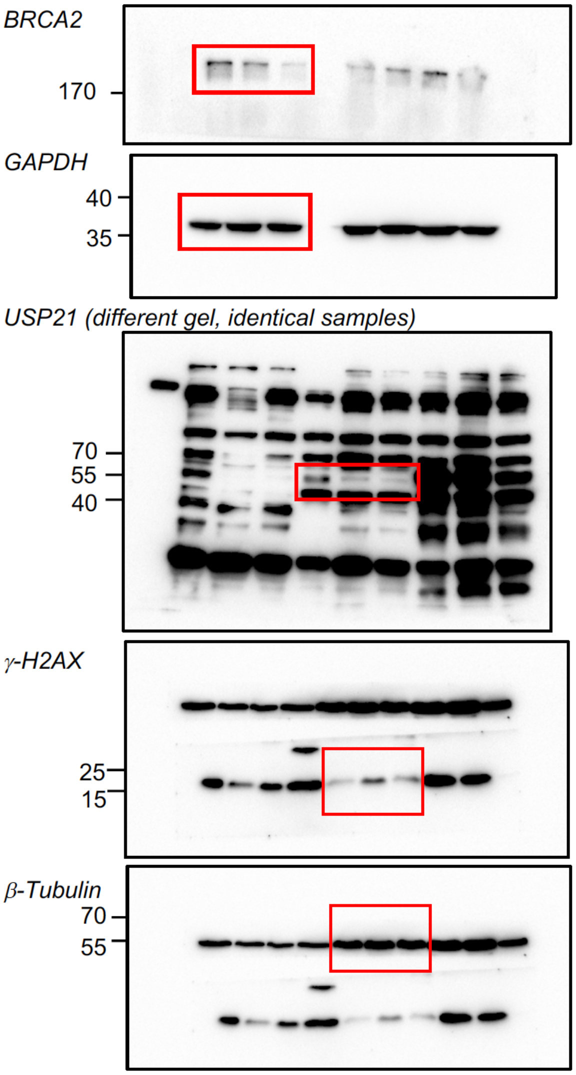

Figure 5B

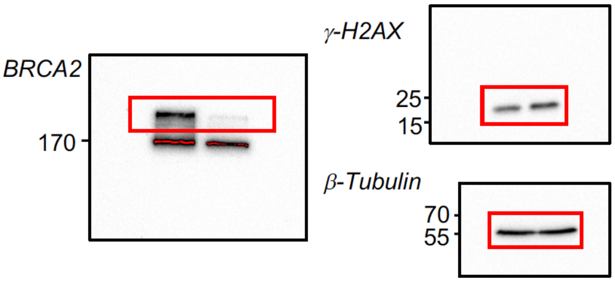

Figure 5E

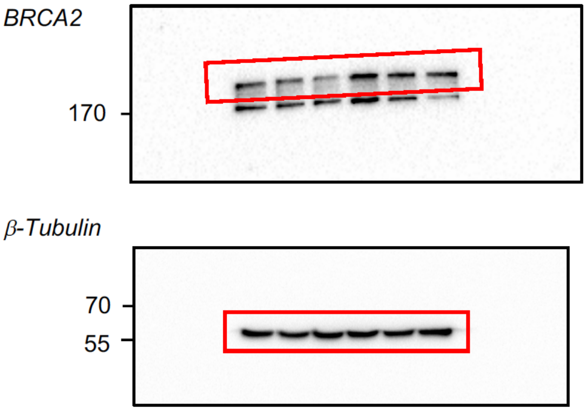

Supplement: Supplementary file 1 — Supplementary Information [file 41467_2017_206_MOESM1_ESM.pdf]
